# New Insights into *Acinetobacter baumannii* Pathogenesis and Therapeutic Implications

**DOI:** 10.3390/pathogens15040391

**Published:** 2026-04-06

**Authors:** Rocco Morena, Helen Linda Morrone, Vincenzo Olivadese, Sara Palma Gullì, Francesca Serapide, Alessandro Russo

**Affiliations:** 1 Infectious and Tropical Diseases Unit, “Renato Dulbecco” Teaching Hospital of Catanzaro, 88100 Catanzaro, Italy; 2 Department of Medical and Surgical Sciences, “Magna Graecia” University, 88100 Catanzaro, Italy

**Keywords:** *Acinetobacter baumannii*, multidrug resistance, carbapenem resistance, biofilm, virulence factors, β-lactam-β-lactamase inhibitors

## Abstract

*Acinetobacter baumannii* is a leading cause of healthcare-associated infections and is classified among the highest-priority antimicrobial-resistant pathogens. Its clinical success reflects the convergence of antimicrobial resistance (AMR) and biological traits that promote environmental persistence and transmission. *Acinetobacter baumannii* has undergone a remarkable transformation over the past few decades, evolving from a relatively obscure environmental bacterium into a globally recognized multidrug-resistant pathogen. Its prevalence in healthcare settings, particularly intensive care units, has made it a leading cause of ventilator-associated pneumonia, bloodstream infections, wound infections, and urinary tract infections. Beyond its antibiotic resistance, the bacterium’s ability to persist in hospital environments and adapt to host defences has amplified its clinical significance. Recent research has uncovered complex networks of virulence factors, regulatory systems, and metabolic strategies that enable *A. baumannii* to thrive in hostile environments and evade host immunity, providing new insights into its pathogenesis and potential therapeutic vulnerabilities. This review summarizes the main mechanisms underlying its pathogenicity, including desiccation tolerance, biofilm formation, disinfectant resistance, metal acquisition, motility, and the ability to enter viable but non-culturable states. In *A. baumannii*, AMR functions as a pathogenesis-adjacent trait, enhancing survival and clonal dissemination through genomic plasticity, resistance islands, efflux systems, and envelope remodeling. Key resistance pathways involve carbapenem-hydrolyzing oxacillinases, metallo-β-lactamases, permeability defects, and multidrug efflux, often coexisting within high-risk clones. From a clinical perspective, management of carbapenem-resistant strains requires accurate infection diagnosis, reliable susceptibility testing, site-specific and PK/PD-optimized therapy, and early reassessment. Overall, the success of *A. baumannii* reflects the integration of resistance and persistence within healthcare ecosystems, highlighting the need for coordinated strategies combining stewardship, infection control, improved diagnostics, and anti-biofilm or anti-virulence approaches.

## 1. Introduction

*Acinetobacter* is a Gram-negative, strictly aerobic, catalase-positive, oxidase-negative non-fermentative coccobacillus, mostly found in soil and water [1]. It comprises around 30 species, several of which can potentially infect humans. The species more frequently reported as human pathogens in literature are *A. baumannii*/*calcoaceticus* complex and *A. iwoffi*, with *A. baumannii* displaying the greatest clinical importance [2,3]. Community acquired infections have mostly been observed in the setting of humid climates, natural disasters and wars, causing outbreaks of fulminant pneumonia and wound infections [4,5]. Nowadays, *A. baumannii* is a leading cause of hospital-acquired infections [6] and poses a major threat in healthcare settings, having been enlisted among the WHO priority pathogens for high levels of antimicrobial resistance [7]. Risk factors for nosocomial infection by *A. baumannii* include admittance to Intensive Care Units (ICUs), mechanical ventilation, exposure to antibiotics, central venous catheterization, recent surgery, rectal or airway colonization, and parenteral nutrition [8,9]. The most common clinical presentations are ventilator-associated pneumonia, catheter-related bloodstream infection (CRBSI), surgical or wound skin and soft tissue infections [10].

Importantly, carbapenem-resistant *A. baumannii* (CRAB) should not be regarded simply as a treatable infection that has acquired resistance, but rather as a hospital-adapted pathogen in which antimicrobial resistance, environmental persistence, and immune evasion converge, sustaining transmission and making eradication particularly challenging in ICU settings [10].

In the present review, we aim to summarize and describe the main physio-pathological features, virulence factors and antimicrobial resistance patterns underlying *Acinetobacter*’s role as a human pathogen.

## 2. Mechanisms of Environmental Persistence

*Acinetobacter baumannii* exhibits an outstanding aptitude to thrive in inhospitable conditions through the coordinated activation of multiple stress-response pathways that promote persistence in hospital environments and reduce susceptibility to decontamination measures. A defining feature of *A. baumannii* is its remarkable resilience under adverse conditions, including prolonged survival on abiotic surfaces, which contributes to environmental persistence and nosocomial transmission [11]. This success relies on a coordinated set of adaptive mechanisms, including surface-associated factors, regulatory systems, and stress-response pathways that enhance survival under desiccation, oxidative stress, and nutrient limitation typical of healthcare environments [12]. Together, these traits allow *A. baumannii* to persist in hospital niches and maintain a stable environmental reservoir for infection.

Recent studies suggest that *A. baumannii* can survive extreme environmental stress by entering a viable but non-culturable (VBNC) state, in which cells remain metabolically active despite loss of routine culturability. This adaptive response has been associated with desiccation and other severe stresses and may contribute to the pathogen’s persistence on hospital surfaces and medical devices despite infection-control measures [13].

### 2.1. Desiccation Endurance

*Acinetobacter*’s intrinsic tolerance to desiccation is among the factors responsible for the persistence of this pathogen in nosocomial settings and abiotic surfaces, where colonisation can lead to opportunistic infections in susceptible human hosts. Survival to very dry environments for extended periods, reaching 2% relative humidity [14], requires a series of adaptive responses to intracellular damage such as accumulation of reactive oxidant species (ROS) with consequent damage to proteins and DNA, disruption of cell membrane permeability and loss of cytoplasmic volume.

The cell capsule plays a key role in desiccation tolerance, as observed in *A. baumannii* strain AB5075, whose colonies are able to switch from opaque to translucent variants: the opaque colonies possess a thicker capsule and show a higher resistance to desiccation and disinfection [15,16].

Biofilm formation, an essential virulence factor, also promotes endurance of *Acinetobacter* to dry conditions by retaining water in the local microenvironment [17]. A gene expression regulating protein BfmR (which is part of the two-component system BfmRS) that appears to be crucial in biofilm formation and desiccation tolerance [18,19] is implicated in the upregulation of genes encoding protective proteins in response to oxidative stress, such as bacterial hydrophilins and catalase KatE [20]. Oxidative stress adaptation is reinforced by dedicated protective circuits, including the manganese-responsive regulator MumR and the OxyR-controlled periplasmic protein HslJ, both of which enhance resistance to reactive oxygen species and support bacterial fitness under hostile conditions [21].

Increased protein aggregation in *A. baumannii* has been associated with desiccation tolerance, suggesting a role of aggregates in protecting the cell proteome from misfolding and degradation due to external stress, although their specific function is yet to be determined [22].

A recent study shows that the complex transcriptional reprogramming of *A. baumannii* cells when undergoing desiccation results in an overall shift in metabolic activity and a transition to a “viable but non culturable” state, which provides an advantage to survival in harsh environments [23].

### 2.2. Disinfection Resistance

Decreased susceptibility of *Acinetobacter* to antiseptic agents commonly used in hospitals raises alarming health concerns and has been linked to selection of multi-drug-resistant strains in healthcare environments [24].

The upregulation of enzymes produced in response to oxidative stress, particularly catalases KatG and KatE, induces resistance to hydrogen peroxide [25]. Low susceptibility to membrane-disrupting antiseptic chlorexidine is conferred by the expression of active efflux pumps known as *Acinetobacter* chlorexidine efflux proteins [26,27]. Other efflux pump encoding genes (quacE, qacEΔ1) are associated with resistance to quaternary ammonium compounds [28,29]. Interestingly, the majority of *A. baumannii* metallo-beta lactamase (MBL) producing strains often harbour these biocide resistance genes, suggesting a possible co-selection of antimicrobial and antiseptic resistance through linkage of genes present on the same mobile elements [30].

Biofilm formation also plays a role in protection against disinfectants; due to its ability to prevent biofilm formation the use of ethylene diamine tetraacetic acid (EDTA) as a synergistic agent combined with an antiseptic agent has been proposed [31].

### 2.3. Virulence Factors

In addition to the mechanisms that enable successful survival in harsh environments, *Acinetobacter* displays a number of virulence factors that are fundamental for host colonization and infection, metabolic adaptation, escape from host immunity and decreased susceptibility to antimicrobials.

*Acinetobacter*, as the Greek derivation of the name suggests (α−κινητο, meaning non-motile), was previously considered a non-motile microorganism due to its lack of flagella. However, studies have proven that *A. baumannii* is indeed capable of movement through two distinct mechanisms: twitching and surface associated motility [32,33].

Twitching is a jerky movement sustained by repeated rounds of extension and retraction sustained by functional type IV pili. Surface-associated motility, also known as swarming motility, describes the cellular ability to move on wet surfaces [34]. While earlier research attributed a key role of IV type pili in this second mechanism, recent studies reveal that movement over wet surface is independent of type IV pili expression and rather relies on chemotaxis, quorum sensing and presence of signaling molecules, among which a unique polyammine named 1,3-diaminopropane (DAP) [35,36]. When this molecule is inactivated by acetylation, cell metabolism tends to switch to a state of decreased motility, adherence and biofilm formation, while increased non-modified DAP levels tip the balance towards planktonic form and motility [37]. Quorum sensing, a complex cell network that influences gene expression according to bacterial cell density, also plays a central role in *A. baumannii* surface-associated motility and biofilm formation, influencing its virulence [38].

### 2.4. Surface Adherence and Biofilm Formation

Biofilms are communities of bacteria encased in an exopolysaccharide matrix that adhere to and colonize both biotic and abiotic surfaces [39]. Transition from a planktonic state to surface adherence and colonization under biofilm formation is an essential virulence factor displayed by *Acinetobacter* spp., particularly when confronting with low nutritional availability. Biofilm production increases pathogen survival in harsh environments by providing resistance to desiccation and disinfection, as mentioned above; it is also an important determinant of resistance to antimicrobial agents in the human host [40].

The first step to biofilm formation is adherence to epithelial cells and abiotic surfaces; this is promoted in *Acinetobacter* by the Csu pili which are formed by the type I chaperone-usher pili assembly system [41]. Genes encoding Csu pili are shown to be significantly overexpressed in biofilm forming colonies, determining early surface colonization and biofilm development on abiotic materials relevant to clinical care, including plastics and metals commonly used in medical devices [42,43]

*A. baumannii* outer membrane protein A (OmpA), a porin embedded in the outer membrane, promotes a variety of virulence mechanisms, including epithelial cell adhesion and intracellular proliferation, biofilm formation, host immune response and host cell apoptosis [44,45]. For this pivotal role in pathogenesis, it has been proposed as a target for treatment of *A. baumannii* infection [46].

Biofilm associated proteins (Bap) are cell wall proteins involved in biofilm production and expressed by a plethora of both Gram positive and negative pathogens. They exhibit a role in maintaining cell hydrophobicity, a feature that enables adherence and biofilm formation [47]. Expression of Bap in *A. baumannii* strains is associated with mature biofilm production, particularly on abiotic surfaces, such as polypropylene and titanium, while Bap-negative isolates show a low biofilm formation activity. Bap gene expression is also associated with multi-drug-resistant strains [48].

Among other genes conferring antimicrobial resistance, blaPER-1, encoding an extended spectrum beta-lactamasis, has also been associated with increased biofilm forming activity [49].

Biofilm maturation is further supported by extracellular matrix components, including poly-β-(1,6)-N-acetylglucosamine (PNAG), which contributes to structural stability of the biofilm matrix and enhances persistence on surfaces [50]. This phenotypic plasticity underscores the sophistication of biofilm-mediated survival strategies in *A. baumannii*.

### 2.5. Metal Acquisition

Most microorganisms require metals such as iron and zinc to survive, as these nutrients are incorporated in many enzymes and proteins that play an important role in maintaining cell homeostasis [51]. Iron is an essential cofactor for many bacterial enzymes such as catalases, cytochromes and succinate dehydrogenases that mediate a number of metabolic functions. Iron and zinc are also involved in DNA repair and replication and in gene expression [52,53]. For this reason, human organisms have adapted their metabolism in order to maintain low levels of circulating iron to withdraw access of this essential element to pathogens, a protection mechanism known as “nutritional immunity” [54]. For example, while most iron in the human body is bound to hemoglobin, other circulating proteins with a high affinity to iron (transferrin, lactoferrin, calprotectin) function as collectors that prevent iron chelation by invading pathogens [55]. Conversely, bacteria express a variety of mechanisms to overcome this protection mechanism and sequester iron and other essential metals from the host [56]. *Acinetobacter* possesses a number of pathways that are activated in response to low iron levels via a metal-regulated transcription factor known as Ferric uptake regulation (Fur) protein [57]. In case of iron deficiency, Fur upregulates the production of siderophores, which are carrier molecules with a high affinity to ferric iron; these are secreted into the extracellular matrix and bind free iron molecules or sequester them from host metal complexes [58]. *A. baumannii* produces three main classes of siderophores: acinetobactins, fimbactins and baumannoferrins. Acinetobactins are the most widely expressed and display a sensitivity to pH: the active form in a high pH environment is acinetobactin while pre-acinetobactin works in acidic conditions [59,60]. Fimsbactins are produced in 10% of strains, fimsbactin A being the most prevalent form; when co-expressed with acinetobactin they appear to have an antagonising effect on this siderophore and *A. baumannii* growth, suggesting a competition of the two proteins for similar pathways [61]. Other bacterial components involved in iron uptake are hemolytic proteins such as phospholipase C that are able to cause erythrocyte lysis and thus increase free iron concentration [61], while some hypervirulent *A. baumannii* strains produce a heme oxygenase that enables heme uptake and metabolization [62]. Mechanisms of zinc sequestration, also essential for bacterial metabolism, are down-or-upregulated in *A. baumannnii* according to its availability via a specific Zinc uptake repressor protein (Zur) [63].

### 2.6. Viable but Non-Culturable State

*A. baumannii* is capable of entering a viable but non-culturable (VBNC) state in response to environmental stresses such as nutrient limitation, desiccation, anaerobiosis, change in temperature and pH, exposure to disinfectants or antibiotics. In this state, bacterial cells remain metabolically active and maintain membrane integrity, while losing the ability to grow on conventional culture media [64,65].

The VBNC state represents an important survival strategy for *A. baumannii*, particularly in hospital environments, where it can persist on abiotic surfaces and medical devices for extended periods. VBNC cells retain virulence-associated traits and may resuscitate under favorable conditions, posing a risk for recurrent infections and outbreaks. Moreover, the presence of VBNC *A. baumannii* may contribute to the underestimation of environmental contamination and infection reservoirs, as standard microbiological methods fail to detect these cells [66].

### 2.7. Evasion of Host Immune Responses

Successful infection requires evasion of the host immune system, and *A. baumannii* has evolved multiple strategies to achieve this. Capsular polysaccharides play a major role in immune evasion by limiting complement deposition and reducing phagocytic clearance, thereby enhancing bacterial survival in host tissues and bloodstream infections [67]. Surface remodeling, including variation in lipooligosaccharide and lipid A structure, can further alter host recognition and decrease susceptibility to cationic antimicrobial peptides, contributing to persistence under immune and antimicrobial pressure [68]. In addition to extracellular defenses, accumulating evidence indicates that some strains of *A. baumannii* can survive within epithelial cells and macrophages, thereby partially escaping extracellular immune surveillance and potentially contributing to persistence during infection [67].

Beyond these structural defenses, *A. baumannii* can also modulate host signaling pathways. OmpA is a key virulence determinant involved in host–cell interaction and injury, while outer membrane vesicles (OMVs) serve as delivery platforms for immunologically active bacterial components, including OmpA itself. Recent literature supports a role for OmpA and OMV-associated activities in altering inflammatory signaling, damaging host cells, and reshaping cytokine responses, although the precise magnitude of these effects may vary across strains and experimental models [44,69]. Together, these mechanisms highlight a complex host–pathogen interplay in which *A. baumannii* not only avoids immune elimination but also actively modifies the host response to prolong survival and facilitate dissemination.

### 2.8. Metabolic Flexibility and Adaptation During Infection

Metabolic plasticity is an important component of *A. baumannii* pathogenesis. Recent genomic, transcriptomic, and metabolic studies indicate that this pathogen can reprogram central carbon metabolism according to nutrient availability and host-imposed stress, allowing growth in nutritionally restricted niches such as blood and the lower respiratory tract. Rather than relying on a single preferred nutrient source, *A. baumannii* can exploit diverse substrates and adjust pathways linked to gluconeogenesis, the tricarboxylic acid cycle, fatty acid metabolism, and amino acid utilization, thereby supporting both fitness and persistence during infection [70]. Consistent with this view, genome-scale metabolic modeling in a murine bloodstream infection model identified major shifts in energy production, central metabolism, and cell envelope biogenesis, suggesting that metabolic rewiring is integral to successful host adaptation [71]. More recently, lactate utilization has emerged as a concrete example of this flexibility: disruption of lactate uptake reduced in vivo fitness and abolished the increased tolerance to killing in human blood observed after growth in biologically relevant lactate concentrations [72].

These adaptations are also highly relevant in the biofilm state. Biofilm-associated *A. baumannii* does not simply grow more slowly, it adopts a distinct physiological program characterized by metabolic heterogeneity, altered respiration, and enhanced tolerance to antibiotics and host defenses [73]. This persistent phenotype is sustained by coordinated regulatory responses rather than by metabolic quiescence alone. In particular, the BfmRS two-component system acts as a central stress-response hub and links environmental sensing to surface persistence programs, including traits associated with biofilm fitness and survival under hostile conditions [12]. In parallel, metabolic pathways can directly influence antibiotic tolerance and virulence, as illustrated by the phenylacetic acid catabolic pathway, which contributes to oxidative-stress adaptation, antibiotic responses, and infection fitness [74]. Overall, these findings support bacterial metabolism as a promising anti-virulence target, especially for pathways that become critical during infection or biofilm growth but are less important in nutrient-rich conditions.

The emergence of small-colony variants (SCVs) has been described as a potential mechanism of bacterial persistence and it is involved in the pathogenesis of biofilm-related infections and chronic infections, thus there are few data regarding *A. baumannii* SCVs and its clinical relevance [75]. Recent evidence suggests the potential role of Vitamin B12 in the selection of *A. baumannii* SCVs with increased MIC to cefiderocol, showing a new additional adaptative trait which could lead to the development of resistance and reduced efficacy to first line antibiotics [76].

### 2.9. Genomic Plasticity and the Interplay Between Resistance and Virulence

*A. baumannii* exhibits marked genomic plasticity, driven by horizontal gene transfer, recombination, and the accumulation of mobile genetic elements such as plasmids, insertion sequences, transposons, and resistance islands. This genomic fluidity helps explain the substantial heterogeneity observed among clinical isolates, whose resistomes and virulomes can vary considerably across sequence types and lineages [73,77]. Accordingly, the relationship between resistance and virulence is better viewed as strain-dependent than uniform: comparative genomic studies suggest that some adaptive determinants may support persistence and pathogenicity, whereas others are associated primarily with antimicrobial resistance, producing different evolutionary balances in different clonal backgrounds [12]. In this context, whole-genome sequencing has become increasingly valuable not only for outbreak investigation and phylogenetic tracking, but also for defining strain-specific combinations of resistance genes, virulence loci, and mobile elements that may influence clinical behavior [12,73].

Among the mechanisms underlying this diversity, horizontal gene transfer appears especially important. Recent work has reinforced the relevance of natural transformation in *A. baumannii*, showing that AbaR-type resistance islands are closely linked to transformation-mediated genomic exchange and that their chromosomal insertion sites can influence their long-term persistence in bacterial populations [71]. In parallel, insertion sequences remain major drivers of resistance evolution. In particular, ISAba1 and related elements can enhance expression or mobilization of adjacent β-lactam resistance determinants, and recent evidence indicates that ISAba1-mediated amplification of intrinsic chromosomal oxacillinase loci may directly confer carbapenem resistance [73]. Overall, the mosaic genome of *A. baumannii* provides a mechanistic basis for the co-evolution of resistance and virulence and supports the growing role of genome-informed approaches in both epidemiology and therapeutic stratification. These persistence mechanisms are not merely ecological traits but have direct clinical implications, as they sustain environmental reservoirs, facilitate recurrent infections, and undermine the effectiveness of infection-control measures, particularly in ICU settings.

## 3. Antimicrobial Resistance as a Driver of Persistence and Pathogenic Success

*Acinetobacter baumannii* illustrates how antimicrobial resistance (AMR) can provide more than antibiotic protection, acting as an ecological advantage that promotes persistence and spread in healthcare settings. This is reflected in international priority lists: carbapenem-resistant *A. baumannii* (CRAB) is included in the highest priority group of the 2024 WHO Bacterial Priority Pathogens List [7] and classified as an urgent threat by the CDC [78]. These designations highlight not only limited therapeutic options, but also the pathogen’s remarkable ability to persist and circulate in hospitals.

In ICU and other high-risk wards, antibiotic and environmental pressures select for lineages in which resistance is tightly linked to persistence and ongoing transmission.

A major contributor to this success is the ability of *A. baumannii* to survive on dry, abiotic surfaces. Desiccation tolerance is an active, regulated process involving stress-response proteins such as hydrophilins that support survival and influence infection-related phenotypes [20]. Environmental stress can also induce profound metabolic downregulation consistent with dormancy-like or VBNC states, allowing resistant populations to evade routine detection while retaining the capacity to resuscitate when conditions improve [66]. This persistence amplifies the clinical impact of AMR by facilitating repeated reintroduction of the same resistant clones.

Genomic plasticity further links resistance with epidemic success. *A. baumannii* readily acquires resistance determinants through insertion sequences, plasmids, and large chromosomal resistance islands, including AbaR structures found in globally disseminated clones [79]. In addition, exposure to subinhibitory or heterogeneous antibiotic concentrations—common in biofilms or contaminated environments—can trigger stress responses and genetic diversification, reinforcing both resistance and persistence [80].

Overall, in *A. baumannii*, AMR should be viewed as a pathogenesis-adjacent trait that enhances environmental survival, clonal stability, and transmission within modern healthcare systems.

### 3.1. β-Lactam Resistance: Enzymes, Expression Platforms, and Target Adaptation

Resistance to β-lactam antibiotics is the most clinically important mechanism driving *A. baumannii* pathogenic success, particularly for carbapenems, which have long been mainstays of therapy for serious infections. Carbapenem resistance in *Acinetobacter baumannii* should be interpreted as a multifactorial phenotype arising from the interplay between β-lactamases, permeability defects, efflux systems, and target modifications, rather than from a single dominant mechanism.

#### 3.1.1. Class D Carbapenem-Hydrolyzing Oxacillinases (CHDLs)

In *Acinetobacter baumannii*, carbapenem resistance is primarily mediated by class D carbapenem-hydrolyzing oxacillinases (CHDLs). The most prevalent acquired enzymes include OXA-23, OXA-24/40, and OXA-58, whereas OXA-51-like enzymes are intrinsic and chromosomally encoded [81].

Alone, OXA-51-like enzymes usually confer low-level activity but provide a baseline that can be enhanced under specific genetic conditions. A key determinant of CHDL-mediated resistance is gene expression control. Many *OXA* genes are associated with insertion sequences, particularly ISAba1, which can provide a strong upstream promoter. This configuration markedly increases transcription and results in clinically significant carbapenem resistance, even in the absence of additional carbapenemases. Notably, ISAba1-driven overexpression may transform intrinsic OXA-51-like enzymes into effective resistance determinants, blurring the boundary between intrinsic and acquired resistance [82,83].

From an epidemiological standpoint, these expression platforms contribute to the global success of CRAB clones. The same OXA enzyme may produce different resistance phenotypes depending on promoter context, gene copy number, and genomic location [81]. Therefore, carbapenem resistance in *A. baumannii* depends not only on enzyme type but also on the genetic architecture regulating its expression, supporting clonal persistence and dissemination in high-pressure hospital environments.

#### 3.1.2. Metallo-β-Lactamases and Other β-Lactamases

Although CHDLs account for most carbapenem resistance in *A. baumannii*, the acquisition of metallo-β-lactamases (MBLs) represents a major clinical concern. New Delhi metallo-β-lactamase (NDM) is the most relevant and increasingly reported enzyme, conferring resistance to nearly all β-lactams and remaining unaffected by currently available β-lactamase inhibitors. NDM frequently coexists with additional resistance mechanisms, leading to extensively drug-resistant phenotypes and severely limiting therapeutic options [84].

Other MBLs, including VIM and IMP, are less commonly detected in *A. baumannii* than in other Gram-negative pathogens but retain significant clinical impact. These enzymes are typically located on mobile genetic elements, facilitating dissemination and increasing the risk of local or regional outbreaks [85].

Beyond carbapenemases, *A. baumannii* possesses the intrinsic *Acinetobacter*-derived cephalosporinase (ADC), a class C β-lactamase contributing to cephalosporin resistance. Similar to CHDLs, ADC expression can be upregulated by upstream insertion sequences, particularly ISAba1, resulting in high-level resistance [82]. Although extended-spectrum β-lactamases (ESBLs) play a less central role than in Enterobacterales, their presence may further increase β-lactam resistance when combined with carbapenemases and permeability defects.

Overall, non-CHDL β-lactamases are less prevalent, but enzymes such as NDM are disproportionately important because of their broad spectrum and major therapeutic implications.

#### 3.1.3. Permeability and PBP

β-lactam resistance in *A. baumannii* is frequently the result of combined mechanisms, in which enzymatic hydrolysis is reinforced by changes in the bacterial cell envelope. Reduced permeability plays a key role: loss or modification of outer membrane proteins and porins—most notably CarO—can limit carbapenem entry into the periplasm and act synergistically with β-lactamases to raise resistance levels. In this context, even modest enzymatic activity can become clinically significant when antibiotic influx is reduced [81].

Target modification also contributes to resistance, particularly in relation to new β-lactam strategies. Sulbactam has intrinsic activity against *A. baumannii* through binding to specific penicillin-binding proteins (PBPs), which has led to the development of combinations such as sulbactam-durlobactam. However, reduced susceptibility to these agents has been associated with alterations in PBP3 and with the presence of certain β-lactamases, including MBLs [86,87]. This highlights that resistance to novel β-lactam combinations may arise not only through enzyme acquisition but also through target adaptation.

### 3.2. Non-β-Lactam Resistance Mechanisms

Beyond β-lactams, *A. baumannii* frequently becomes resistant to polymyxins, tetracyclines, aminoglycosides, and fluoroquinolones. These mechanisms are clinically relevant because they often appear in the same high-risk clones and can interact with persistence traits (envelope adaptation, stress tolerance, and biofilm-associated survival), further limiting treatment options [88]. The main non-β-lactam resistance mechanisms are summarized in Table 1.

### 3.3. Efflux Pumps and Envelope Adaptations as “Cross-Cutting” Determinants

Efflux pumps and envelope adaptations represent cross-cutting determinants in *Acinetobacter baumannii*, as they simultaneously affect susceptibility to multiple antibiotic classes and enhance survival under hospital-relevant stresses. In a pathogenesis-oriented framework, systems that reduce intracellular drug accumulation or limit drug entry also support persistence on devices and surfaces, allowing resistant lineages to remain successful even when antibiotic pressure fluctuates.

Among efflux mechanisms, tripartite RND-family pumps (notably AdeABC, AdeIJK, and AdeFGH) are key contributors to multidrug resistance and part of broader adaptive responses [99]. Efflux activity is also linked to tolerance to non-antibiotic stressors. Chlorhexidine and quaternary ammonium compounds can induce efflux gene expression, and pump inhibition reduces tolerance to these biocides, demonstrating a role in survival during disinfectant exposure [100]. In addition, long-chain polyamines are substrates of the disinfectant efflux pump AmvA, and AdeABC contributes to polyamine tolerance, highlighting the ability of major efflux systems to export diverse toxic compounds beyond antibiotics [101].

Envelope adaptations act synergistically with efflux. Changes in outer-membrane permeability, lipopolysaccharide structure, and porin expression reduce antibiotic influx while increasing tolerance to disinfectants, desiccation, and oxidative stress, thereby promoting environmental persistence in healthcare settings. Envelope remodeling further enhances survival outside the host under environmental stressors [99,102]. Overall, efflux and envelope modifications form an integrated module linking multidrug resistance with environmental resilience and long-term transmission in hospital ecosystems.

### 3.4. Cefiderocol and Emerging Resistance Under Therapeutic Pressure

Cefiderocol is a siderophore cephalosporin that exploits iron-uptake pathways and shows significant activity against carbapenem-resistant *A. baumannii*. However, resistance may emerge during therapy through a combination of reduced drug uptake, β-lactamase background, and target-related changes. Decreased susceptibility often reflects multiple concurrent mechanisms, including β-lactamases, alterations in TonB-dependent siderophore receptors, and modifications affecting PBP3, supporting a multifactorial rather than single-gene resistance model [103].

Among β-lactamases, PER-type enzymes (and to a lesser extent NDM) contribute to reduced cefiderocol susceptibility, indicating that the pre-existing resistance background can increase MIC values and facilitate selection under treatment [104]. Impaired uptake represents another common pathway to resistance. Treatment-emergent resistance has been associated with reduced expression of TonB-dependent receptors such as PiuA, limiting intracellular drug accumulation [105]. These findings support evidence that alterations in iron-transport systems can compromise cefiderocol’s “Trojan horse” mechanism, with therapeutic pressure selecting for reduced entry rather than classical β-lactamase-mediated resistance.

Despite these mechanistic insights, resistance to cefiderocol remains overall limited and is typically associated with specific resistance backgrounds or treatment-emergent adaptations rather than widespread dissemination [105,106].

Clinically, heteroresistance is an additional concern, as resistant subpopulations may expand during exposure and lead to failure despite baseline susceptibility [107]. Host- and environment-related factors may also influence resistance development. Vitamin B12 has been shown to modulate bacterial metabolism, promoting reduced susceptibility and the emergence of small-colony variants in CRAB [75]. Overall, cefiderocol remains a valuable option, but its activity can be compromised by uptake alterations, resistance background, and adaptive selection, underscoring the need for careful susceptibility testing and surveillance [106].

## 4. Treatment of Infections Caused by CRAB

Infections caused by carbapenem-resistant *Acinetobacter baumannii* (CRAB) primarily affect critically ill patients and are associated with high mortality, prolonged hospitalization, and limited therapeutic options. Treatment is further complicated by frequent comorbidities, delayed appropriate therapy, and the need to balance efficacy with toxicity in severely unstable clinical settings [108].

Pathogenesis in *A. baumannii* is strongly niche-dependent, with different host environments selecting different virulence programs. In the respiratory tract, infection is typically associated with a robust innate inflammatory response, especially neutrophil recruitment, but capsule- and surface-associated defenses help the bacterium withstand complement and phagocyte-mediated clearance. In invasive infection, disease severity is shaped not only by bacterial burden but also by host innate immune signaling, with TLR4, inflammasome-related pathways, and complement contributing to outcome variability in experimental models. This context dependence helps explain why clinical manifestations range from localized pneumonia to fulminant sepsis, and why host factors substantially influence susceptibility and progression [109,110,111].

These pathogenic insights have stimulated interest in anti-virulence and host-directed strategies. Rather than directly killing the organism, these approaches aim to disarm traits such as biofilm formation, capsule-associated protection, iron acquisition, or OmpA/OMV-mediated host interaction, thereby reducing selective pressure for conventional resistance. In parallel, immunologic approaches remain under active investigation: OmpA is one of the best-studied targets for antibody- and vaccine-based intervention, while capsular and outer-membrane antigens continue to be explored as candidate immunogens [112]. Bacteriophage therapy is another promising adjunct, particularly in combination with antibiotics, where recent work supports phage–antibiotic synergy against multidrug-resistant *A. baumannii* and biofilm-associated infection [113]. Together, these approaches reflect a shift from exclusively bactericidal therapy toward strategies informed by host–pathogen biology and bacterial persistence mechanisms.

### 4.1. Clinical Framework and Key Principles

Management of CRAB infections should begin with clinical-microbiological confirmation of true infection rather than colonization, particularly in respiratory and urinary samples. Treatment decisions should consider (i) infection site and achievable drug exposure, (ii) severity (sepsis or shock), (iii) need for source control (devices or collections), and (iv) timely, reliable susceptibility testing [114,115]. Observational studies of CRAB bloodstream infection and bacteremic VAP report high mortality, and early effective therapy appears critical, supporting rapid escalation to an active regimen in unstable patients and prompt de-escalation once infection is excluded or controlled [116,117].

A practical bedside approach stratifies cases into: (A) severe invasive disease (BSI, septic shock, or bacteremic VAP) and (B) localized infection with lower bacterial burden. Category A generally requires optimized dosing and, when supported by evidence, combination therapy or a high-barrier agent, whereas category B may allow narrower regimens once susceptibility is confirmed [114,115]. In all cases, PK/PD constraints must be assessed (e.g., lung penetration, protein binding, renal function, CRRT), as underexposure may lead to microbiological failure and resistance selection.

Laboratory results require cautious interpretation. Colistin testing remains method-dependent, and cefiderocol susceptibility may be affected by methodological variability and heteroresistance, supporting close clinical monitoring and repeat cultures in non-responding patients [75,114]. A stewardship approach is essential: avoid treating colonization, shorten therapy when response is rapid, and use follow-up cultures selectively in high-risk invasive infections.

Overall, CRAB management should be infection-confirmed, site-specific, PK/PD-optimized, and guided by reliable AST, with early source control and reassessment within 48–72 h [75,114,115,116,117].

### 4.2. Sulbactam-Durlobactam-Based Regimens

When available, sulbactam-durlobactam is the most evidence-supported first-line option for severe CRAB infections. Its rationale is biologically grounded: sulbactam has intrinsic activity against *A. baumannii* through binding to key penicillin-binding proteins, but this effect is often compromised by the β-lactamase background typical of CRAB. Durlobactam, a diazabicyclooctane inhibitor active against Ambler class A, C, and D serine β-lactamases–including OXA-type enzymes–was developed to protect sulbactam and restore effective target engagement [118].

Microbiological data support this combination, showing potent in vitro activity against geographically diverse isolates of the *A. baumannii-calcoaceticus* complex, including multidrug-resistant strains [119]. Clinically, sulbactam-durlobactam has demonstrated efficacy comparable to colistin-based regimens in terms of 28-day mortality, with significantly improved renal safety. These findings support its preferential use over polymyxins, particularly in critically ill patients where nephrotoxicity is a major concern [120]. Current international guidelines include sulbactam-based therapy (preferably combined with a β-lactamase inhibitor) among the preferred strategies for invasive CRAB infections, emphasizing optimized dosing and early reassessment in unstable patients [114,115].

Limitations should be considered. Durlobactam lacks activity against MBLs, and reduced efficacy has been associated with specific resistance backgrounds or alterations affecting sulbactam targets, such as PBP modifications, highlighting the need for susceptibility testing and close clinical monitoring when response is suboptimal [118,121]. Overall, sulbactam-durlobactam represents a preferred backbone for severe CRAB infections, combining a targeted mechanism with stronger clinical evidence and improved tolerability compared with historical standards [114,115,120].

### 4.3. Alternatives When Sulbactam-Durlobactam Is Unavailable

When sulbactam-durlobactam is unavailable, therapy should follow a structured approach balancing severity, infection site, and reliable susceptibility data, recognizing the heterogeneity of available evidence. Current guidance supports high-dose sulbactam exposure as the treatment backbone, typically combined with at least one additional active agent in severe invasive infections [114,115].

High-dose ampicillin-sulbactam is a practical option, exploiting sulbactam’s intrinsic activity against *A. baumannii*. In severe ventilator-associated pneumonia, high-dose regimens have shown outcomes comparable to polymyxin-based therapy [122], and combinations (e.g., with colistin) have been associated with improved clinical responses compared with colistin alone in ICU cohorts [123]. Polymyxins are therefore generally reserved as alternative or rescue agents due to nephrotoxicity and the need for close monitoring, but they remain important when few active options are available [114,115].

Cefiderocol may be considered when susceptibility is confirmed and clinical context supports its use, particularly to avoid polymyxin toxicity. Real-world data in severe CRAB infections, including ICU pneumonia and bacteremia, suggest favorable outcomes in selected patients, although interpretation is limited by confounding and heterogeneous background regimens [117]. Given the risk of resistance emergence and heteroresistance, its use requires close clinical reassessment and follow-up cultures in non-responders [114,115].

Minocycline or tigecycline can serve as partner agents when susceptibility is documented, most often in combination regimens for pneumonia or soft-tissue infections, with minocycline preferred when PK and breakpoints are favorable. Overall, a practical hierarchy is (i) high-dose ampicillin-sulbactam combinations for severe disease, (ii) cefiderocol for selected susceptible isolates, and (iii) polymyxin-based regimens when alternatives are lacking, with early reassessment and source control [114,115].

### 4.4. Role of Cefiderocol

Cefiderocol is a siderophore cephalosporin with preserved in vitro activity against many CRAB isolates and may represent a valuable option when first-line therapies are unavailable, not tolerated, or inactive. However, its clinical positioning requires careful patient selection. In a randomized comparison against best available therapy for carbapenem-resistant infections, a signal of higher mortality was observed in the subgroup with *Acinetobacter* infections, leading to a more cautious interpretation of its role in severe disease [106].

Subsequent observational studies in critically ill patients suggest that cefiderocol-containing regimens may achieve favorable outcomes in selected cases, including severe pneumonia and bloodstream infection, although these findings should be interpreted with caution due to confounding and heterogeneity in background therapy [117,124,125]. Current guidance therefore recommends considering cefiderocol primarily when susceptibility is confirmed and alternative preferred options are not feasible [114,115].

An additional concern is the potential for resistance emergence during therapy, often related to alterations in iron-transport pathways or the underlying β-lactamase background. For this reason, cefiderocol use should be accompanied by close clinical reassessment and repeat microbiological evaluation in patients with delayed or suboptimal response [114,115].

### 4.5. Polymyxins: Current Role and Toxicity

Polymyxins (colistin and polymyxin B) were historically central to CRAB therapy but are now considered alternative or salvage options when preferred agents are unavailable or inactive. Their use is limited by variable efficacy in severe infections and unfavorable pharmacokinetics, including suboptimal lung penetration and a narrow therapeutic window. Pharmacokinetics are complex, and nephrotoxicity is frequent in critically ill patients, particularly with prolonged treatment; therefore, close renal monitoring and early reassessment are essential. Given unpredictable clinical response and the risk of resistance emergence, polymyxins are generally reserved for salvage use, often within combination regimens for severe infections when no better options exist [114,115,126].

Accurate susceptibility testing is critical. For colistin, broth microdilution is the reference method, and EUCAST has repeatedly warned against unreliable commercial methods, such as gradient tests, which may produce misleading MICs and inappropriate treatment decisions [127]. Overall, polymyxins should be used only when alternatives are limited, with careful monitoring and early de-escalation when feasible.

### 4.6. Tetracyclines and Other “Partner Drugs”

Tetracycline derivatives may be used as adjunctive agents for CRAB infections when preferred options are unavailable or when additional active therapy is required. Their role is limited by bacteriostatic activity and the need to achieve adequate site exposure, making careful patient selection essential. Current guidance supports high-dose minocycline or tigecycline mainly within combination regimens, particularly when in vitro susceptibility is confirmed and alternatives are limited [114,115].

Minocycline is often preferred due to the availability of intravenous and oral formulations and a more favorable pharmacokinetic profile, although achieving effective exposure may be challenging at higher MIC values. Tigecycline may be considered in selected cases, but susceptibility breakpoints for *A. baumannii* are inconsistent, and standard dosing has been associated with suboptimal outcomes in severe infections; therefore, high-dose regimens and combination therapy are recommended when used [113,114,128].

Overall, tetracyclines should be considered partner agents rather than backbone therapy, reserved for selected cases with confirmed susceptibility and favorable pharmacokinetic conditions [113,114,129].

### 4.7. Combination Therapy vs. Monotherapy: What the Evidence Supports

In CRAB infections, the choice between combination therapy and monotherapy should be guided by evidence quality, infection severity, and the agent’s resistance barrier rather than a routine escalation approach. The strongest randomized data come from a trial in severe *Acinetobacter* infections showing that a modern sulbactam-based regimen achieved outcomes comparable to colistin with significantly less nephrotoxicity, supporting the use of a validated backbone rather than empirical multidrug therapy [120]. Accordingly, current guidance prioritizes selecting the most active, best-supported agent first, reserving combination therapy for specific situations [114,115].

Outside sulbactam-durlobactam, evidence for combination regimens remains inconsistent. For polymyxins, randomized and observational studies have not consistently demonstrated a survival benefit, and reported advantages are often confounded by differences in severity, source control, and background therapy. In ventilator-associated pneumonia, adding high-dose ampicillin-sulbactam to colistin has been associated with improved clinical outcomes compared with colistin alone, although the generalizability of this strategy remains uncertain [123]. Consequently, guidelines discourage routine polymyxin monotherapy in severe invasive infections but emphasize that combinations should be purposeful–for example, to increase the likelihood of at least one active agent, address borderline susceptibility, or manage high-inoculum infections–rather than automatic [114,115].

In practice, monotherapy is appropriate when a preferred active agent with reliable susceptibility and adequate site exposure is available. Combination therapy is most justified in septic shock, high-burden infections (e.g., bacteremic VAP), uncertain susceptibility or AST limitations, or when using agents with narrow therapeutic windows or a higher risk of resistance emergence [114,115].

### 4.8. Site-Specific Considerations

Site of infection should guide agent selection and expectations of efficacy. For HAP/VAP, ensure adequate lung exposure and avoid agents with unreliable epithelial lining fluid concentrations; high bacterial burden and biofilm on ventilator circuits favor early optimization and strict source control. For bloodstream infection/sepsis, prioritize rapidly bactericidal, well-supported regimens and reassess within 48–72 h with repeat cultures in unstable patients. In intra-abdominal infection, source control is often decisive and antibiotics should be tailored once drainage is achieved. For skin/soft tissue, debridement and shorter courses may be feasible when response is prompt. For central nervous system infection, penetration becomes the key limiting factor and options are restricted. For urinary isolates, confirm true infection and avoid treating colonization. These principles are consistent across current international guidance [114,115].

### 4.9. Diagnostic and Stewardship Takeaways

Effective CRAB management requires diagnostic accuracy and strict stewardship. First, confirm that the isolate reflects true infection rather than colonization, especially in respiratory and urinary samples, and reassess therapy within 48–72 h based on clinical course, source control, and microbiological data [114,115]. Second, the quality of susceptibility testing is critical: colistin MICs should be determined using reference methods (broth microdilution), as EUCAST has warned about the unreliability of several commercial assays [127]. Similar caution applies to cefiderocol, for which EUCAST has reported accuracy and reproducibility issues with some commercial tests and recommends specific MIC methodologies and confirmatory testing when results are uncertain [130,131].

Follow-up cultures should be used selectively, mainly in high-risk invasive infections (e.g., persistent bacteremia or ongoing sepsis) or when clinical response is delayed, to identify microbiological failure or emerging resistance. Finally, limit unnecessary antibiotic exposure: avoid treating colonization, de-escalate when narrower active options are available, optimize dosing to prevent underexposure, and keep treatment duration as short as clinically appropriate once stability and source control are achieved [114,115]. These measures reduce toxicity, limit resistance selection, and improve assessment of clinical response in critically ill patients.

## 5. Conclusions and Future Perspectives

*Acinetobacter baumannii* is a clear example of how AMR and biological persistence converge to sustain transmission in healthcare settings, particularly for carbapenem-resistant lineages prioritized by international agencies [7,76]. Beyond resistance, the pathogen’s success is supported by desiccation tolerance, biofilm formation, and adaptive stress responses, which allow survival on abiotic surfaces and medical devices and promote recurrent outbreaks [11,12,13,14,15,16,17,18,19,35,36]. VBNC-like states further complicate detection and may contribute to underestimation of reservoirs and delayed control [64,65,66]. Genomic plasticity, including resistance islands and mobile elements, links resistance acquisition with clonal expansion under sustained hospital pressure [79,80].

Therapeutically, the field is shifting toward evidence-supported backbones such as sulbactam–durlobactam for severe CRAB infections, offering efficacy with improved safety compared with historical standards [118]. However, emerging resistance and testing limitations—particularly for colistin and cefiderocol—reinforce the need for reliable AST and close clinical reassessment [75,114,115,127,130,131,132,133]. Future work should prioritize anti-biofilm/anti-virulence strategies and diagnostics that capture persistence phenotypes alongside resistance, integrating stewardship with infection prevention to reduce selection and transmission [114,115].

**Figure 1:**
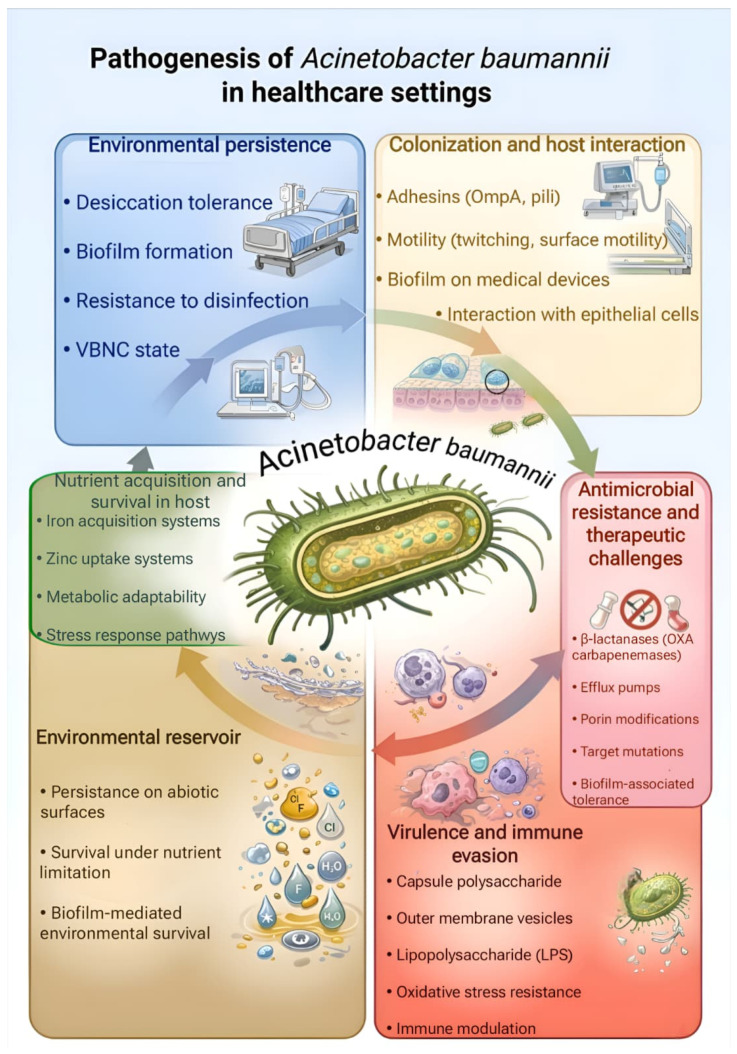
Pathogenesis of *Acinetobacter baumannii* infections in healthcare settings. This figure was created by the authors with the assistance of AI-based tools (Google Gemini, version 1.5 Pro) and has not been previously published.

The interplay between environmental persistence, virulence mechanisms, and antimicrobial resistance that underlies the clinical success of *A. baumannii* in healthcare settings is schematically summarized in Figure 1.

## Figures and Tables

**Table 1:** Non-β-lactam resistance mechanisms in *Acinetobacter baumannii* and their pathogenesis implications.

Antibiotic Class	Main Resistance Mechanism	Key Determinants	Pathogenesis/Persistence Relevance	Clinical Implications	References
Non-β-lactam agents (overview)	Multimechanism resistance frequently co-occurring within high-risk clones	Efflux systems, envelope adaptation, target mutations, mobile genetic elements	Mechanisms overlap with stress tolerance, biofilm survival, and environmental persistence, reinforcing MDR/XDR phenotypes	Markedly reduced therapeutic options and increased risk of treatment failure	[88,89]
Polymyxins	Lipid A modification with reduced surface charge	pmrCAB mutations or regulatory activation → phosphoethanolamine addition to lipid A	Maintains LPS structure and bacterial fitness, supporting long-term persistence and epidemiological success	Stable resistance emerging under polymyxin pressure	[90]
Polymyxins	Complete loss of lipopolysaccharide (target loss)	Inactivating mutations in lipid A biosynthesis genes (lpx pathway)	Associated with fitness costs (membrane instability, reduced virulence), potentially limiting long-term dissemination	Often selected under intense antibiotic pressure; biologically unstable phenotypes	[91,92]
Tetracyclines (tigecycline, minocycline)	Active efflux via RND pumps	Overexpression of AdeABC (often due to AdeRS mutations); contribution of AdeIJK and AdeFGH	Low fitness cost and broad substrate profile support adaptation to heterogeneous exposures (e.g., biofilms, devices) and environmental stress tolerance	Reduced susceptibility, particularly to tigecycline; requires susceptibility-guided dosing and combination strategies	[93,94,95]
Aminoglycosides	Enzymatic drug modification and target protection	Aminoglycoside-modifying enzymes; acquisition of 16S rRNA methyltransferases	Genes located on mobile elements facilitate co-selection with other resistance determinants and stabilization within epidemic clones	High-level, class-wide resistance frequently embedded in MDR lineages	[88,96,97]
Fluoroquinolones	Target-site mutations in quinolone resistance-determining regions (QRDR)	Chromosomal mutations in gyrA and parC	Stable chromosomal resistance maintained during clonal expansion, consolidating MDR phenotypes	Further restriction of combination options, especially in severe infections	[98]

Abbreviations and acronyms: RND: resistance–nodulation–division family efflux pumps. QRDR: quinolone resistance–determining region. LPS: lipopolysaccharide. MDR: multidrug-resistant. XDR: extensively drug-resistant.

## Data Availability

No new data were created or analyzed in this study. Data sharing is not applicable to this article.
